# Concept of a point of care test to detect new oral anticoagulants in urine samples

**DOI:** 10.1186/1477-9560-11-15

**Published:** 2013-08-01

**Authors:** Job Harenberg, Sandra Krämer, Shanshan Du, Christel Weiss, Roland Krämer

**Affiliations:** 1 Clinical Pharmacology, Medical Faculty of Medicine Mannheim, Ruprecht-Karls-University Heidelberg, Heidelberg, Germany; 2 Biometry and Statistics, Medical Faculty of Medicine Mannheim, Ruprecht-Karls-University Heidelberg, Heidelberg, Germany; 3 Inorganic Chemistry Institute, Ruprecht-Karls-University Heidelberg, Heidelberg, Germany

**Keywords:** Oral anticoagulant, Dabigatran, Rivaroxaban, Apixaban, Renal function, Anticoagulation, Urine, Coagulation assay, Monitoring, Compliance

## Abstract

New oral anticoagulants (NOAC) are approved for several indications for prophylaxis and treatment of venous thromboembolism and for prevention of embolism in atrial fibrillation at fixed daily doses without need of laboratory guided dose adjustment. Due to their low molecular weight of about 500 to 600 Dalton and their hydrophilicity free anticoagulant is excreted immediately through glomerular filtration into the urine. Impairment of renal function may increase the plasma concentration of the anticoagulants and lowered creatinine clearance is a declared contraindication. In contrast to the initial aim of development the anticoagulant effect is required to be determined in special clinical situations. Several specific and non-specific assays using plasma samples are currently undergoing standardization. As all NOACs are excreted into the urine, specific assays were developed for this matrix to determine them quantitatively of qualitatively. Urine samples can be easily and repetitively obtained avoiding problems and risks associated with blood sampling. The qualitative assay can be performed as a point of care test (POC) also by the patient by judging the different colours for the absence or presence of the drugs with the naked eye. The test is rapid (results available within 15 min), sensitive, specific and accurate and does not require a purified NOAC as control. The tests may be a tool for clinicians who need to know for treatment decisions if a NOAC is on board or not. As the tests are specific for oral direct thrombin inhibitors and for oral direct factor Xa inhibitors, the indication does not interfere with other qualitative POC test in development using clotting systems. The test may be indicated for patients at acute hospitalization, before surgery or central nervous system puncture anaesthesia, if fibrinolytic therapy is indicated, acute deterioration of renal function, and for control of adherence to therapy.

## Introduction

Thromboembolic complications are one of the major complications following primary elective total hip (THR) and knee replacement (TKR) surgery with considerable morbidity and mortality, which can be reduced substantially by subcutaneous low-molecular weight heparins and new oral anticoagulants [1]. Cerebral and non-cerebral embolism is the most relevant severe event occurring in patients non-valvular atrial fibrillation (AF) which can be effectively prevented by vitamin-K antagonists (VKA) [2]. Limitations of the conventional regimes for prophylaxis of venous thromboembolism (VTE) with low molecular weight heparin (LMWH) include local haematoma and allergy, heparin-induced thrombocytopenia type I and type II, transient increase of liver enzymes, and the requirement for parenteral administration [3]. VKA requires frequent dose adjustments to obtain the time in the therapeutic range of international normalized ratio (INR) values between 2 and 3 [4]. Many interactions with food and drugs, the slow onset and offset of action of VKAs require simultaneous administration of UFHs or LMWHs in many clinical situations. Severe intracranial and extracranial bleeding complications and other severe side effects also limit the administration of VKA. The underuse of vitamin-K antagonists is one of the consequences of the fear of bleeding complications especially in older [5]. One option to improve the efficacy and safety of treatment with VKA is to adopt point-of-care whole blood devices for self-testing and self-management [6-8]. However, this option is limited to a small group of patients able to follow the instruction of the POC device and restricted by the unwillingness of health insurance systems to cover the additional expenses [9]. To improve and facilitate oral anticoagulant therapy small molecular orally available anticoagulants (NOAC) specifically inhibiting factor Xa or thrombin were developed and some of them are now approved for several indications.

NOAC do not require routine drug monitoring to adjust the dose because they have a relatively low variation of their pharmacological profile after administration in man. Because all NOACs are small molecules with a molecular weight of about 500 to 600 dalton, renal function plays a major role for the metabolism ranging from 80% for the direct thrombin inhibitor dabigatran to about 33% for factor Xa inhibitors rivaroxaban [10] and 25% for apixaban. Accordingly, reduction of creatinine clearance to less than 30 ml/min for dabigatran or 15 ml/min for rivaroxaban and apixaban are regarded as a contraindication for treatment. The dose of dabigatran has to be reduced by about 30% according to the European guidelines and by 50% according to the food and drug administration guidelines for creatinine clearance between 50 and 30 ml/min, and the doses of rivaroxaban and apixaban by 50% for creatinine clearances between 30 and 15 ml/min.

The concentration of NOACs can be accurately determined by high-pressure liquid chromatography methods. These techniques are, however, unsuitable for clinical routine use. Thrombin and factor Xa dependent coagulation or chromogenic substrate assays have therefore been developed to respectively determine the concentration or activity of dabigatran and rivaroxaban [11,12]. However, point of care (POC) methods to determine NOACs have not been reported so far. Measurement of anticoagulants in urine has been reported previously by the authors for heparin [13], a modified heparin derivative [14], r-hirudin and its pegylated derivative [15], metabolites of phenprocoumon [16] and of fibrinopeptide A, which is a split product of fibrinogen upon action of thrombin [17].

Advantages of urine sampling include the non-invasive techniques and the possibility to develop POC test systems. These assays can be performed by trained personal or by the patients themselves, and can be repeated easily [18,19].

### Indications for determination of NOACs

In specific clinical situations determination of NAOCs may be required for clinical decision making. Typical examples, where it is necessary to know if the drug is on board or not, are indication for fibrinolytic therapy in cerebral embolism, placement of a lumbar catheter, and severe bleeding events.

Renal function reduces with increasing age. Many diseases do or may reduce renal function independently of age. Therefore, renal elimination of NOACs is reduced in various clinical conditions (Table 1).

**Table 1:** Clinical situations where determination of NOACs may be required

•	Before surgery, lumbar anesthesia
•	At any hospitalization
•	Any bleeding/haemorrhage
•	Any thrombotic and embolic event
•	Indication for thrombolytic therapy
•	Suspicion of deterioration of renal function
•	Dehydration / exsiccation
•	Newly diagnosed pregnancy
•	Very elderly
•	Children
•	Adherence to therapy /compliance
•	Suspicion of intoxication

### Rationale of the POC test from urine

Development of point of care (POC) methods for NOACs in urine is based on their immediate glomerular filtration and excretion into the urine when they are present in blood. This concept was used to develop specific POC methods for oral direct factor Xa inhibitors and oral direct thrombin inhibitors.

## Methods

**Figure 1:**
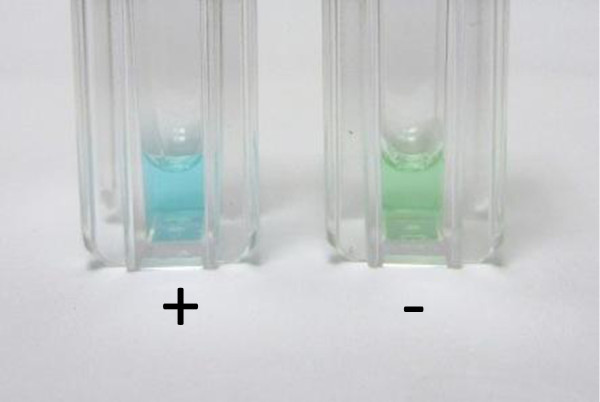
Results of the POC test with urine of a patient not on treatment with dabigatran (left, blue colour) and on treatment with dabigatran (right, green colour).

**Figure 2:**
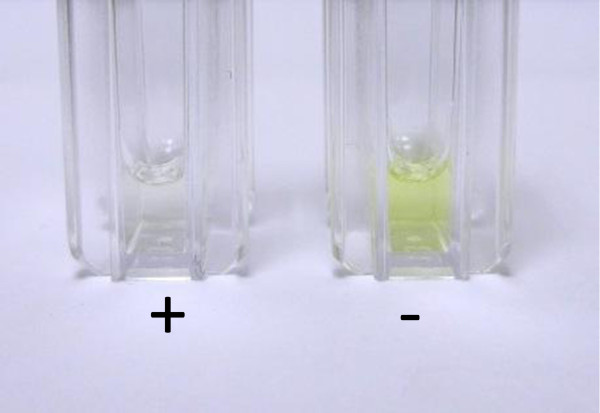
Results of the POC test with urine of a patient not on treatment with rivaroxaban (left, transparent colour) and on treatment with rivaroxaban (right, yellow, no colour).

The test principle is based on the development of different colours in the presence and absence of oral direct factor Xa and thrombin inhibitors. The details of the methods are described in the patent applications for direct thrombin inhibitors [20] and direct factor Xa inhibitors [21]. For a POC testing it becomes important that the colours are read correctly by the naked eye. Mistakes should not occur for identification of the colours. This has been taken into consideration for the development of the test systems. Accordingly, colours for negative and positive were chosen with a contrast high enough to differentiate between presence and absence of the NOAC. Importantly, colour for factor Xa and thrombin inhibitors had to be different to avoid a confounding of two classes of inhibitors. Two colours were chosen for each class of inhibitors: for factor Xa (absence: yellow colour, present: clear colour, Figure 1) and thrombin inhibitors (absence: green colour, present: blue colour, Figure 2) are different.

As described [19], dabigatran was incubated with ministrips containing lyophilized reagents followed by incubation with patient’s urine [20]. The initially blue colour of the solution changes to a green colour in the absence of dabigatran within 15 minutes.

In detail [19], rivaroxaban was determined by a POC method incubating the lyophilized reagents on strips followed by incubation with patient’s urine over 15 min [21]. The yellow colour of the urine itself is diluted with the reagents to a transparent solution. If the urine does not contain rivaroxaban, a yellow colour develops during incubation with the reagents immobilized on the strips. If the urine contains rivaroxaban no colour develops and the solution remains clear.

## Results

The test results of the determination of dabigatran and rivaroxaban are shown in Figures 1 and 2. To determine the correctness of identifying the colours by patients with the naked eye, positive and negative samples for dabigatran and rivaroxaban were prepared and were read by patients [19]. The results of the sensitivity, specificity, accuracy, positive and negative predictive indices are given in Table 2.

**Table 2:** Results of the sensitivity, specificity, accuracy, positive predictive value, and negative predicted value of the POC test from urine samples of patients treated with dabigatran or rivaroxaban

	**Dabigatran N=484**		**Rivaroxaban N=457**	
	**Mean**	**95% CI**	**Mean**	**95% CI**
Sensitivity	100	99–100	96	94–98
Specificity	99	98–100	98	96–99
Accuracy	99	99–100	97	96–98
PPI	99	98–100	98	97–99
NPI	100	99–100	96	94–98

*PPI* positive predictive index, *NPI* negative predictive index. Results of patients not on treatment with anticoagulants served as control (adapted from 19).

The results show that the values are somewhat higher with the dabigatran test using green and blue colours. The results with rivaroxaban a little bit worse, but still very high with values for sensitivity, specificity and accuracy all above 95%. Importantly, the yellow colour of normal urine is diluted in the test procedure and do not influence the results. However, if a urine sample is very concentrated the dilution of the yellow colour may not be sufficient and may lead to intermediate colour which cannot be clearly adjudicated as transparent or yellow. Detailed results of the POC method in urine were published earlier [18,19].

## Discussion

In certain clinical situation may require the determination of their anticoagulant effect. These situations may occur more frequently in acute than in chronic situations. POCT methods are available and are successfully used for determination of the INR during treatment with VKA. At present, the TP reagents used for the currently available POCT monitors are not sensitive towards rivaroxaban or dabigatran (unpublished data). In chronic therapy adherence to drug treatment was shown to increase by using POCT methods [22]. POCT methods from urine samples have been reported for several drugs [23], and have shown to improve adherence to therapy [24]. Urine samples are easy to collect and are commonly used to control adherence to drug therapy. Samples can be collected by the patient and any health care personal. If samples are analysed by a POCT system, results are available within minutes. The strength of the methods is that they are not invasive, repetitively to perform, that results are available within 15 min, that patients can perform the test themselves, and the tests do not require standards as control. The methods are sensitive, specific, accurate and posses a very high inter-rater agreement [19].

Limitations of the test system for both test system are, that they become negative if renal impairment is reduced to more than 10 ml/min creatinine clearance. This value may be more relevant for dabigatran than the oral direct factor Xa inhibitors because of the higher elimination rate into the urine. A limitation of the test for dabigatran exists for patients with a red-green amblypoia. Relatives or other persons have to perform the test. A limitation for the oral direct factor Xa inhibitor test is that concentrated urine may produce a yellow colour. To avoid such misinterpretation, a scale with colours of native urine has to be added to the test system indication that the testing is possible or not possible.

Other limitations of the POC methods include the lack of information about the compliance of the patient. Patients may not have taken the last dose of the NOAC before blood collection and urinary excretion may still continue. This is particularly essential for emergency physicians, who should be aware on how to interpret results. However, acute urine samples can be repetitively obtained in emergency situations to cover this problem.

In conclusion, determination of dabigatran, rivaroxaban and other direct factor Xa inhibitors from urine using POC methods are characterized by a high sensitivity, specificity, and accuracy. The POC methods are non-invasive, easy to perform and can be done repetitively. The use in real world situations remains to be investigated [19].

## Competing interests

The authors do not have any conflict of interest and do not have to declare any financial or non-financial interest regarding the manuscript.

## Authors’ contributions

Contributions to conception and design were made by JH, RK, and CW. Acquisition, analysis and interpretation of data were performed by SK, SD, and CW. For drafting the manuscript or revising it critically for important intellectual content participated JH, RK, SK, and SD. All authors have given final approval of the version to be published.
